# Comparison of 3-Dimensional and Augmented Reality Kidney Models With Conventional Imaging Data in the Preoperative Assessment of Children With Wilms Tumors

**DOI:** 10.1001/jamanetworkopen.2019.2633

**Published:** 2019-04-19

**Authors:** Lianne M. Wellens, Jene Meulstee, Cornelis P. van de Ven, C. E. J. Terwisscha van Scheltinga, Annemieke S. Littooij, Marry M. van den Heuvel-Eibrink, Marta Fiocco, Anne C. Rios, Thomas Maal, Marc H. W. A. Wijnen

**Affiliations:** 1 Department of Pediatric Surgery, Princess Máxima Center for Pediatric Oncology, Utrecht, the Netherlands; 2 Radboud University Medical Center, Nijmegen, the Netherlands; 3 Department of Radiology, University Medical Centre Utrecht, Utrecht, the Netherlands; 4 Division of Medical Statistics, Department of Biomedical Data Sciences, Leiden University Medical Centre, Leiden, the Netherlands; 5 Mathematical Institute, Leiden University, Leiden, the Netherlands

## Abstract

**Importance:**

Nephron-sparing surgery can be considered in well-defined cases of unilateral and bilateral Wilms tumors, but the surgical procedure can be very challenging for the pediatric surgeon to perform.

**Objective:**

To assess the added value of personalized 3-dimensional (3-D) kidney models derived from conventional imaging data to enhance preoperative surgical planning.

**Design, Setting, and Participants:**

In a survey study, the conventional imaging data of 10 Dutch children with Wilms tumors were converted to 3-D prints and augmented reality (AR) holograms and a panel of pediatric oncology surgeons (n = 7) assessed the quality of the different imaging methods during preoperative evaluation. Kidney models were created with 3-D printing and AR using a mixed reality headset for visualization.

**Main Outcomes and Measures:**

Differences in the assessment of 4 anatomical structures (tumor, arteries, veins, and urinary collecting structures) using questionnaires. A Likert scale measured differences between the imaging methods, with scores ranging from 1 (completely disagree) to 5 (completely agree).

**Results:**

Of the 10 patients, 7 were girls, and the mean (SD) age was 3.7 (1.7) years. Compared with conventional imaging, the 3-D print and the AR hologram models were evaluated by the surgeons to be superior for all anatomical structures: tumor (median scores for conventional imaging, 4.07; interquartile range [IQR], 3.62-4.15 vs 3-D print, 4.67; IQR, 4.14-4.71; *P* = .008 and AR hologram, 4.71; IQR, 4.26-4.75; *P* = .002); arteries (conventional imaging, 3.62; IQR, 3.43-3.93 vs 3-D print, 4.54; IQR, 4.32-4.71; *P* = .002 and AR hologram, 4.83; IQR, 4.64-4.86; *P* < .001), veins (conventional imaging, 3.46; IQR 3.39-3.62 vs 3-D print, 4.50; IQR, 4.39-4.68; *P* < .001 and AR hologram, 4.83; IQR, 4.71-4.86; *P* < .001), and urinary collecting structures (conventional imaging, 2.76; IQR, 2.42-3.00 vs 3-D print, 3.86; IQR, 3.64-4.39; *P* < .001 and AR hologram, 4.00; IQR, 3.93-4.58; *P* < .001). There were no differences in anatomical assessment between the two 3-D techniques (the 3-D print and AR hologram).

**Conclusions and Relevance:**

In this study, the 3-D kidney models were associated with improved anatomical understanding among the surgeons and can be helpful in future preoperative planning of nephron-sparing surgery for Wilms tumors. These models may be considered as a supplementary visualization in clinical care.

## Key Points

**Question:**

What is the value of 3-dimensional (3-D) visualization methods, augmented reality holograms, and 3-D printing for the preoperative assessment of anatomical structures in children with Wilms tumors?

**Findings:**

In this survey study of 7 pediatric surgeons for which 10 personalized, augmented reality holograms and 3-D prints of children with Wilms tumors were created, there was a significant added value from the 3-D visualization methods compared with conventional imaging for the preoperative assessment.

**Meaning:**

Three-dimensional visualizations may provide added value for the pediatric surgeon in the anatomical assessment of Wilms tumors, may help in future preoperative planning of nephron-sparing surgery, and may be considered an innovative supplementary visualization in clinical care.

## Teaser

This study compares conventional imaging, augmented reality holograms, and 3-dimensional printing of kidney models to assess the added value of each imaging method for preoperative evaluation of children with Wilms tumors.

## Introduction

Wilms tumors (WTs) are the most frequently occurring pediatric cancers of the kidney. The survival rate of children with WT is around 90%,^1,2,3^ yet approximately 5% of the cases present with bilateral disease, which reveals an overall survival rate of approximately 80%.^4^

In the presentation of bilateral disease, nephron-sparing surgery is the preferred or recommended treatment of choice. Compared with unilateral tumors, bilateral disease carries a higher risk for end-stage renal disease (12%) and secondary morbidity.^4,5^ The benefit of nephron-sparing surgery in unilateral WT is debatable. The excellent survival of patients with unilateral WT has motivated investigation into reducing treatment morbidity while preserving survival by considering nephron-sparing surgery.^6,7^ To reduce the probability of long-term kidney function loss, to reduce the occurrence of perioperative complications, and to facilitate complete tumor resection, a personalized planning and surgical strategy is essential.

Magnetic resonance imaging (MRI) and computed tomography (CT) are used for diagnosis and to differentiate between tumor and healthy renal tissue. Pediatric surgeons plan the surgery of WT based on the 2-dimensional (2-D) interpretation of these conventional imaging techniques. The use of 3-dimensional (3-D) visualizations is hoped to further improve the understanding of the exact tumor location and the assessment of relevant anatomical structures, such as arteries, veins, and urinary collection structures. Data from MRI and CT can be used, possibly fused, and reconstructed into 3-D visualizations.^8^

The technique of 3-D visualization is gradually gaining potential in many surgical disciplines and can be used to define the optimal surgical strategy.^9,10,11,12^ These new techniques can even further improve the assessment of the relevant anatomy and enhance preoperative surgical planning. The 3-D printing of organs and structures has proved valuable for multiple disciplines within the engineering field and clinical practice, such as urology, neurosurgery, cardiac surgery, plastic surgery, and maxillofacial surgery.^12,13,14^^,^ However, 3-D printing is not the current standard of care. In addition, augmented reality (AR) is a technology that can visualize virtual 3-D objects in the real world. The implementation of AR is promising as a supplementary operating tool; its value currently is being assessed in different medical specialties.^15^

In this study, we compared the use of two 3-D visualization techniques—3-D printing and AR—for optimizing the surgical planning of nephron-sparing surgery for WT. To our knowledge, the added value of different 3-D visualization methods in addition to conventional imaging in children with cancer has never before been investigated. A panel of pediatric oncology surgeons in the Netherlands was asked to evaluate the 3-D visualization techniques and report about the potential added value of their use before surgery.

## Methods

### Population

Imaging data from 10 patients diagnosed with a WT in the Princess Máxima Center for Pediatric Oncology, Utrecht, the Netherlands, between January 1, 2016, and May 1, 2017, were included in our study. Patients with metastases at diagnosis were excluded, and 3 patients presenting with bilateral WTs were selectively included. Seven other patients were selected based on best-quality conventional imaging available. Data from conventional imaging (MRI and/or CT scans) were derived from these 10 selected patients. In most patients, MRI was the preferred imaging technique for diagnosis and optimal tumor assessment. Computed tomography was performed to clarify vascular anatomy when nephron-sparing surgery was likely to be performed. The Medical Research Involving Human Subjects Act did not apply to this study, and we received official approval from the medical research ethics committees of the University Medical Center Utrecht, Utrecht, the Netherlands. All data were deidentified; therefore, it was not necessary, according to Dutch Law, to ask for informed consent. This study followed the Standards for Quality Improvement Reporting Excellence (SQUIRE) reporting guideline for quality improvement.^16^

### Imaging Methods

Contrast-enhanced MRI of the abdomen was performed on a 1.5-T MRI system (Achieva; Philips Medical Systems). Coronal 3-D, T2-weighted imaging along with fat-suppressed T1-weighted imaging before and after the administration of gadolinium-based contrast medium was acquired.^17^ Computed tomography was performed with the 16-row multiple detector CT (Brilliance 16P; Philips Medical Systems). All patients received 1.5 mL/kg of contrast medium, with a maximum of 120 mL scanned in the arterial phase (injection rate of 2 mL/s, with saline solution pushed through the injection line immediately after the injection of the contrast bolus [injection rate of 2 mL/s and a volume of 8-10 mL depending on the age of the patient]) in accordance with standard protocol. Exposure settings were adjusted to patient size (range, 104-150 mA and 80-90 kV[p]). Thin section images were reconstructed with 0.90-mm thickness and stored in a 512 × 512 data matrix.

### 3-D Segmentation

The MRI or CT scans from each patient were loaded as digital imaging and communications in medicine (DICOM) files and segmented by an information technology expert from Materialise of Leuven, Belgium. After consulting the pediatric radiologist (A.S.L.) and a pediatric surgeon (C.P.v.d.V.), the correct anatomical segmentations were generated based on the conventional imaging by using Mimics Innovation Suite 3-D segmentation software, version 20 (Materialise).

Each anatomical structure (kidney parenchyma, tumor, arteries, veins, and kidney urinary collecting structures) was segmented separately and given a different color. After the full segmentation was completed, small windows were cut out of the kidney parenchyma, allowing a full view of the tumor and its border separated from the healthy tissue, intrarenal vasculature, and urinary collecting structures. Segmentations were saved as a stereolithography file (.STL) that was suitable for 3-D printing and AR.

### 3-D Printing

The 3-D models were printed using 3-D printing technology (Z Corporation) at Materialise. The printer deposits a liquid binder onto thin layers of powder via the ink-jet printheads, which reacts with an agent in the powder to create a solid, multicolor 3-D model.

### Augmented Reality

A mixed reality headset (HoloLens; Microsoft Corp) was used for AR visualization. The headset uses a head-mounted display with a stereo see-through display and a wireless design. This composition provides a realistic 3-D image and stimulates the user to inspect holograms from different positions and view angles. The spatial impression enables physicians to analyze complex anatomical structures in an interactive way and enhances their perspective of the surgical site.^18^ First, from the exported 3-D models, minor artifacts were repaired in proprietary 3-D software (MeshMixer; AutoDesk Inc). The mesh density of every 3-D model was optimized and consisted of 2000 to 15 000 triangles depending on the size of the models. This process guaranteed a clear visualization on the headset without losing quality. Second, the Unity 3-D software framework, version 5.6.5 (Unity Technologies) was used to create an application for the headset to visualize the kidney, tumors, and relevant anatomy in 3 dimensions. Voice instructions were implemented to rotate, adjust, or manipulate the anatomical 3-D objects. Visualization options were created to make structures transparent, look inside the kidney, separate the tumor from the kidney, and zoom in on specific structures. Although each 3-D model was different and unique to each patient’s tumor and organ anatomy, the architecture and user interface of the application were similar across the sample group. Finally, the application was imported to the headset. An example of a patient’s WT is provided in the Video.

**Video.** Use of 3-D Printed Models and Augmented Reality Holograms for Preoperative Visualization and Planning 3-D visualization is becoming popular among surgeons as a way of planning for procedures. This video illustrates augmented reality (AR) hologram visualization of a child’s Wilms tumor through a HoloLens, Microsoft’s mixed reality headset. By aiming the cursor (floating yellow circle in the video) on the structures of preference, the AR viewer can make the modeled structures transparent, look inside the kidney, zoom in on specific structures, or separate the tumor from the kidney.

### Data Acquisition

A panel of 7 experts (C.P.v.d.V., C.E.J.T.v.S., and M.H.W.A.W.), consisting of 6 pediatric oncology surgeons and 1 pediatric urologist with oncology experience, were asked to individually evaluate the MRI and/or CT images before surgery for every patient. Expertise of the 7 surgeons varied from less than 1 year to 30 years. Two-dimensional images were shown using a DICOM viewer, version 2.4.1 (The Horos Project). Afterward, the surgeons completed a questionnaire regarding the quality of this conventional imaging on the visualization of the anatomical structures in the kidney. Surgeons were asked to score the visibility of the 4 anatomical structures: tumor, arteries, veins, and urinary collecting structures from 1 to 5 (1 indicates completely disagree; 2, disagree; 3, neutral; 4, agree; and 5, completely agree). Scores were requested for the conventional imaging (MRI and/or CT) and for the 3-D visualizations (3-D print and AR), as well as to assess the decision making of the surgeons to perform nephron-sparing surgery or a nephrectomy and their preoperative preparation. The questionnaires are provided in the eAppendix in the Supplement.

Next, the 3-D print of the corresponding patient was provided to the members of the panel. They were asked to complete a second questionnaire with the same questions used for the conventional imaging techniques but with supplementary questions about the added value of the 3-D print in assessing the tumor, artery and venous structures, and urinary collecting structures.

The mixed reality headset was then introduced to visualize the AR hologram of the corresponding patient, and the same questions used to assess the 3-D print were given in the third questionnaire. The above events took place in 1 session per expert for a maximum of 3 hours used to score all 3 modalities for the 10 patients. The cycle of questionnaires for conventional imaging, 3-D print, and AR was repeated for every patient, resulting in the opinions of the 7 experts about all 10 patients.

### Statistical Analysis

Statistical analyses were performed using GraphPad prism, version 7 (GraphPad Software). To compare scores on the MRI and/or CT, 3-D print, and AR hologram, the nonparametric-grouped, Wilcoxon matched-pair signed rank test was used. The Mann-Whitney test was used to compare the difference between questions about the 4 anatomical structures for the individual patients. A 2-sided *P* < .025 (adjusted for multiple testing) was used to test for a significant difference between the conventional imaging and the 3-D visualizations.

## Results

### Patient Characteristics and 3-D Visualizations

**Figure 1.**
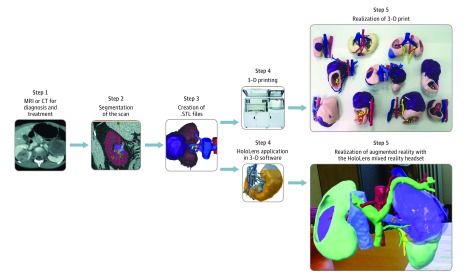
Workflow Diagram Depicting the Construction Process of 3-Dimensional (3-D) Visualizations From the patient-derived magnetic resonance image (MRI), computed tomographic (CT) image, or both, a corresponding 3-D print and augmented reality hologram was made. In step 3, segmentations were saved as stereolithography (.STL) files.

Of the 10 patients, 7 were girls, and the mean (SD) age was 3.7 (1.7) years. The kidney, WT, arteries, veins, and urinary collecting structures were reconstructed as shown in Figure 1. For patient 2, the urinary collecting structures were not reconstructed because of insufficient conventional imaging. The characteristics of the 10 patients are summarized in Table 1.

**Table 1.** Patient Characteristics

Patient No./Sex/Age, y	Type of Wilms Tumor	Preoperative Conventional Imaging Available
1/M/2	Unilateral	MRI
2/M/2	Bilateral	MRI/CT
3/M/3	Bilateral	MRI/CT
4/F/2	Unilateral	MRI
5/M/4	Bilateral	MRI/CT
6/F/3	Unilateral	MRI
7/F/5	Unilateral	MRI
8/F/5	Unilateral	MRI
9/F/7	Unilateral	MRI
10/F/4	Unilateral	MRI

Abbreviations: CT, computed tomography; MRI, magnetic resonance imaging.

**Figure 2.**
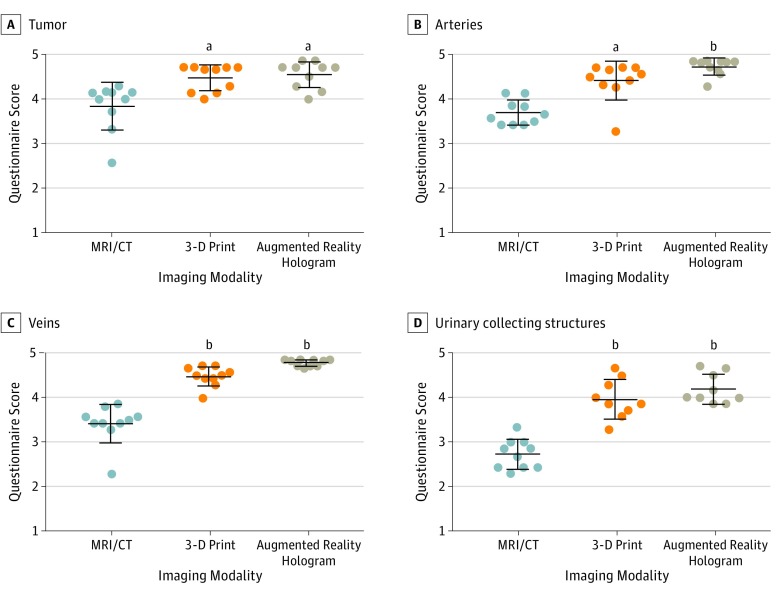
Questionnaire Results About Conventional Imaging, 3-Dimensional (3-D) Prints, and Augmented Reality (AR) Holograms Surgeons scored the visibility of the 4 anatomical structures from 1 to 5 (1 indicates completely disagree; 2, disagree; 3, neutral; 4, agree; and 5, completely agree) for conventional imaging (magnetic resonance imaging [MRI] and/or computed tomography [CT]) and for the 3-D visualizations (3-D print and AR holograms), with results showing the comparison of means of 10 patients. Center lines indicate the medians; error bars, the interquartile ranges. ^a^*P* < .01 compared with MRI/CT. ^b^*P* < .001 compared with MRI/CT.

For all 4 anatomical structures, the 3-D print and the AR hologram received higher scores compared with the conventional imaging (Figure 2). When scores for the 4 anatomical structures were compared between the 3-D print and the AR hologram, no difference was found (Figure 2).

### Assessment of All 4 Anatomical Structures

Both 3-D print and AR holograms led to better assessment of the tumor, arteries, veins, and urinary collection structures compared with conventional imaging (Figure 2 and Table 2) (tumor: median scores for conventional imaging, 4.07; interquartile range [IQR], 3.62-4.15 vs 3-D print, 4.67; IQR, 4.14-4.71; *P* = .008 and AR hologram, 4.71; IQR, 4.26-4.75; *P* = .002; arteries: conventional imaging, 3.62; IQR, 3.43-3.93 vs 3-D print, 4.54; IQR, 4.32-4.71; *P* = .002 and AR hologram, 4.83; IQR, 4.64-4.86; *P* < .001; veins: conventional imaging, 3.46; IQR, 3.39-3.62 vs 3-D print, 4.50; IQR, 4.39-4.68; *P* < .001 and AR hologram, 4.83; IQR, 4.71-4.86; *P* < .001; and urinary collecting structures: conventional imaging, 2.76; IQR, 2.43-3.00 vs 3-D print, 3.86; IQR, 3.64-4.39; *P* < .001 and AR hologram, 4.00; IQR, 3.93-4.58; *P* < .001). There was no significant difference between conventional imaging and 3-D printing or AR in individual cases. Data from all individual cases and the corresponding assessment of the pediatric surgeons is shown in the eFigure in the Supplement.

**Table 2.** Results of the Survey Among 7 Pediatric Surgeons

Anatomical Structure	Score, Median (IQR)		
	MRI/CT	3-D Print	AR Hologram
Tumor	4.07 (3.62-4.15)	4.67 (4.14-4.71)	4.71 (4.26-4.75)
Arteries	3.62 (3.43-3.93)	4.54 (4.32-4.71)	4.83 (4.64-4.86)
Veins	3.46 (3.39-3.62)	4.50 (4.39-4.68)	4.83 (4.71-4.86)
Urinary collecting structures	2.76 (2.42-3.00)	3.86 (3.64-4.39)	4.00 (3.93-4.58)

Abbreviations: AR, augmented reality; CT, computed tomography; 3-D, 3-dimensional; IQR, interquartile range; MRI, magnetic resonance imaging.

### 3-D Kidney Model for Nephron-Sparing Surgery

**Figure 3.**
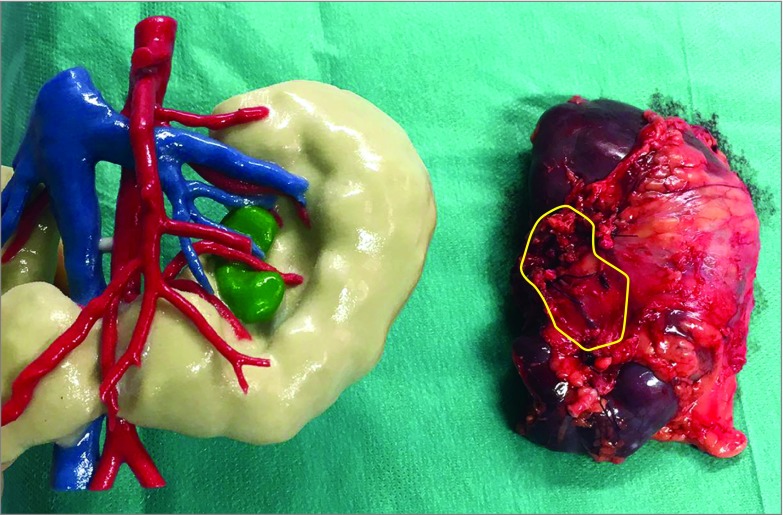
Wilms Tumor 3-Dimensional Print and Corresponding Kidney The yellow outline of the kidney specimen indicates the tumor location.

In 9 of 10 patients, the 3-D print and the AR model were created for research purposes and evaluated after the treatment had been administered. For patient 2, the 3-D model was printed 1 week before surgery because there was a bilateral tumor in a horseshoe kidney. The pediatric surgeons used the model as an assisting tool because of the abnormal anatomical vasculature. A surgical assistant held the 3-D printed model for real-time guidance during surgery (Figure 3).

## Discussion

Preoperative imaging is paramount in achieving good results during oncologic surgery. In nephron-sparing surgery for WT, the risk of positive resection margins is high^6,19^; therefore, it is necessary to improve the present procedure. As opposed to bilateral WT, nephron-sparing surgery in unilateral WT is still debatable, but surgeons recently showed a higher interest in its use for unilateral WT^6,7^ to preserve long-term renal function. However, the use of nephron-sparing surgery is reported to lead to incomplete tumor resection in 30% of unilateral cases, which results in reoperation and additional radiotherapy.^6^ The novel 3-D visualization techniques presented here may be a useful added tool when planning nephron-sparing surgery in unilateral and bilateral WT. In this study, we constructed personalized, high-quality physical and AR 3-D models of pediatric unilateral and bilateral WT to create practice objects for preoperative planning.

To our knowledge, we were the first to reconstruct MRIs and/or CT images of kidneys of 10 patients with WTs in both 3-D prints and AR holograms. We found a reported added value of both of our 3-D models in association with the preoperative assessment by surgeons of the 4 anatomical structures (tumor, arteries, veins, and urinary collecting structure). These data are consistent with previous studies that used 3-D prints in adults with renal cell carcinoma.^20,21^

Detailed understanding of the surgical anatomy of WTs and the surrounding anatomical renal structures in children can be a challenge based on standard 2-D conventional imaging visualizations alone. The lack of ionizing radiation exposure and the improved soft-tissue contrast makes the MRI an attractive imaging method in children.^22^ However, when only MRI is performed, the challenge lies in the accurate detection of the vasculature in these small children. This challenge is particularly true with complex renal anatomy, which often is the case in children with WTs. This study indicated that new, preoperative 3-D imaging processing strategies helped increase the surgeons’ knowledge about the kidney anatomy, thus assisting in improving the planning of tumor resection and optimizing the procedure of choice: nephron-sparing surgery or nephrectomy. This method could improve radicality of tumor resection and spare healthy kidney tissue, thereby preserving long-term renal function.

Converting the existing conventional imaging data to 3-D visualization contributes to overall anatomical understanding.^23^ In this study, no differences were found between the type of 3-D visualization; therefore, the choice between 3-D printing and AR reconstruction may be based on personal preference. Of the multiple 3-D printing techniques, we used the Z-Corp technology, which is able to print accurate models while using different colors. A solid printed model may occlude relevant anatomy, such as blood vessels in the kidney or tumor. To overcome this obstacle, an opening window or transparent material may be used to provide a view inside the model.^24^ Before the start of this study, we printed examples of kidneys with different techniques. We found that the opening window in the solid Z-Corp model best clarified the anatomy. The cost for these multicolored, 3-D printed models was typically $500 (US dollars) and had a manufacturing time of 4 to 5 days. In comparison, the production of an AR reconstruction takes 1 to 2 hours and is, apart from the labor time, costless after initial hardware costs of $3000 to $5000 (US dollars) for the mixed reality headset. The relatively short lead time between chemotherapy and surgery can be an important advantage of the headset. This advantage might make AR reconstruction more feasible and preferable in certain cases. In addition, the AR hologram represented an adaptive and interactive technology compared with the 3-D print in which every structure can easily be opened, switched to transparent, or moved away by giving a voice command.

The wireless design of the mixed reality headset and the use of voice commands for interaction creates the additional possibility of using the headset during surgery.^25^ The AR visualization can be consulted in the operation theater to visualize the anatomy, diseased portion, and 3-D vascularization. In the future, the AR holograms can be fused with the real anatomy of the patient to create a mixed reality setting, as previously described in neurosurgery.^26^ However, more research is needed on how to fuse the virtual holograms with the real nonrigid and deformable anatomy of young patients.

### Limitations

Challenges still exist for the clinical application of the proposed novel 3-D visualization techniques. Conventional medical imaging techniques produce a large amount of information, but good interpretation of these data requires years of expertise. Diffusion-weighted imaging in MRI already shows the increasing potential to discriminate tumor from healthy tissue.^17^ To further objectify these data with scientific accuracy to convert them into 3-D prints or AR holograms, more research about standardized algorithms is needed. In addition, high-quality conventional imaging (MRI or CT) is key for obtaining useful preoperative 3-D imaging. We had to exclude several cases based on insufficient imaging quality. There is a need for more research into standardizing the optimal imaging method (MRI, CT, or CT angiogram), section thickness, and timing for contrast enhancement, specifically in children of different ages. In the present study, the segmentation was performed manually in close collaboration between an information technology expert, a pediatric radiologist, and a pediatric oncology surgeon. Standardizing this process may save valuable time for medical experts.

## Conclusions

This study suggests that 3-D visualization may have an added value for surgeons in the preoperative assessment of children with WT. Additional understanding of the anatomy by using 3-D technology was found for all 4 anatomical structures (the tumor, arteries, veins, and urinary collecting structures). Future research should be aimed at improving the speed, accuracy, and automation of the segmentation process for the 3-D visualization and expanding its clinical use in pediatric oncologic surgery.

## Notes

Supplement. **eAppendix.** Questionnaire 3-D–Imaging Techniques **eFigure.** Outcome of the Questionnaires of Pediatric Oncology Surgeons on Conventional Imaging, 3-D Prints, and Augmented Reality Holograms Visualized on the HoloLens per Case
