# Supplementary material for: Comparison of 3-Dimensional and Augmented Reality Kidney Models With Conventional Imaging Data in the Preoperative Assessment of Children With Wilms Tumors
Source: JAMA Netw Open. 2019 Apr 19;2(4):e192633. doi: 10.1001/jamanetworkopen.2019.2633 (PMC6481457; doi:10.1001/jamanetworkopen.2019.2633)
Supplement: Supplement. — eAppendix. Questionnaire 3-D–Imaging Techniques eFigure. Outcome of the Questionnaires of Pediatric Oncology Surgeons on Conventional Imaging, 3-D Prints, and Augmented Reality Holograms Visualized on the HoloLens per Case [file jamanetwopen-2-e192633-s001.pdf]

Supplementary Online Content

Wellens LM, Meulstee J, van de Ven CP, et al. Comparison of 3-dimensional and augmented reality kidney models with conventional imaging data in the preoperative assessment of children with Wilms tumors. *JAMA Netw Open*. 2019;2(4):e192633. doi:10.1001/jamanetworkopen.2019.2633

eAppendix. Questionnaire 3-D–Imaging Techniques

eFigure. Outcome of the Questionnaires of Pediatric Oncology Surgeons on Conventional Imaging, 3-D Prints, and Augmented Reality Holograms Visualized on the HoloLens per Case

This supplementary material has been provided by the authors to give readers additional information about their work.

© 2019 Wellens LM et al. *JAMA Network Open*.

eAppendix. Questionnaire 3-D–Imaging Techniques

Conventional imaging in bilateral Wilms tumours

Which pre-operative imaging technique is provided?

- o CT
- o MRI
- o Both

	Strongly disagree Strongly agree
The tumor(s) can be well assessed based on this scan	1 – 2 – 3 – 4 – 5
The arterial structures can be well assessed based on this scan.	1 – 2 – 3 – 4 – 5
The venous structures can be well assessed based on this scan.	1 – 2 – 3 – 4 – 5
The urinary collecting structures can be well assessed based on this scan.	1 – 2 – 3 – 4 – 5
Partial nephrectomy of the left kidney is an option based on this scan.	1 – 2 – 3 – 4 – 5 – not relevant
Partial nephrectomy of the right kidney is an option based on this scan.	1 – 2 – 3 – 4 – 5 – not relevant
This scan prepares me for surgery.	1 – 2 – 3 – 4 – 5
I expect no complicating events based on this scan.	1 – 2 – 3 – 4 – 5
I am likely to consult this scan during surgery.	1 – 2 – 3 – 4 – 5

Comments on the scan:

………
………
………

© 2019 Wellens LM et al. *JAMA Network Open*.

3-Dimensional imaging in bilateral Wilms tumours

3D imaging technique that is being assessed:

- ○ 3D-print
- ○ AR reconstruction

Please select the number below that represents your opinion about the 3D-model for each statement.

	Strongly Disagree — *Strongly Agree*
The tumor(s) can be well assessed based on this model	1 – 2 – 3 – 4 – 5
The arterial structures can be well assessed based on this model.	1 – 2 – 3 – 4 – 5
The venous structures can be well assessed based on this model.	1 – 2 – 3 – 4 – 5
The urinary collecting structures can be well assessed based on this model	1 – 2 – 3 – 4 – 5
Partial nephrectomy of the left kidney is an option based on this model.	1 – 2 – 3 – 4 – 5 – not relevant
Partial nephrectomy of the right kidney is an option based on this model.	1 – 2 – 3 – 4 – 5 – not relevant
This model is of additional value to the current imaging techniques in the preoperative planning.	1 – 2 – 3 – 4 – 5
This model is of additional value to the current imaging techniques in assessing the arterial structures.	1 – 2 – 3 – 4 – 5
This model is of additional value to the current imaging techniques in assessing the venous structures	1 – 2 – 3 – 4 – 5
This model is of additional value to the current imaging techniques in assessing the urinary collecting system	1 – 2 – 3 – 4 – 5
This model is of additional value to the current imaging techniques in assessing the tumour(s)	1 – 2 – 3 – 4 – 5
This model prepares me for surgery.	1 – 2 – 3 – 4 – 5
I expect no complicating events based on this model.	1 – 2 – 3 – 4 – 5
I am likely to consult this model in the pre-operative planning.	1 – 2 – 3 – 4 – 5
I am likely to consult this model during surgery.	1 – 2 – 3 – 4 – 5

© 2019 Wellens LM et al. *JAMA Network Open*.

Comments on the 3D model:

………
………
………

What is your opinion about the use of 3D-models for pre-operative planning?

………
………
………

What is your opinion about the use of 3D-models during surgery?

………
………
………

© 2019 Wellens LM et al. *JAMA Network Open*.

eFigure. Outcome of the Questionnaires of Pediatric Oncology Surgeons on Conventional Imaging, 3-D Prints, and Augmented Reality Holograms Visualized on the HoloLens *per Case*

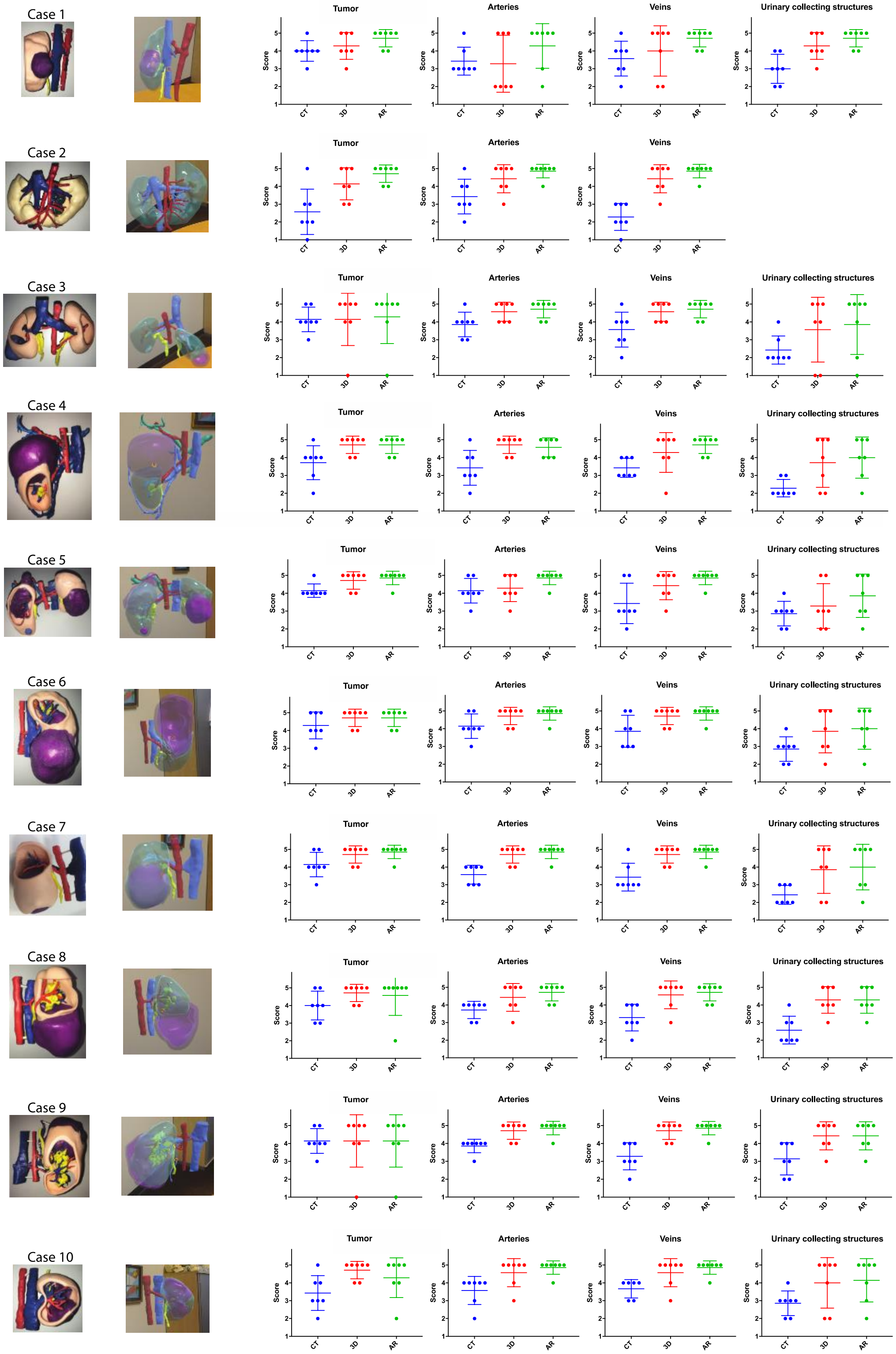

© 2019 Wellens LM et al. *JAMA Network Open*.

eFigure. Surgeons were asked to score the visibility of the four anatomical structures: tumor, arteries, veins and urinary collecting system from 1 to 5 (1=completely disagree, 2 = disagree, 3=neutral, 4=agree, 5=completely agree). Scores were asked for the conventional imaging (MRI and/or CT), and for the 3D visualizations (3D print and AR). This figures shows the ten different patient cases and the corresponding comparison of medians for the three imaging modalities for assessment of the tumor, arteries, veins and urinary collecting structures. In case 2, the urinary collecting structures were not visualized due to insufficient conventional imaging quality. The center lines indicate the medians of the score of the ten patients. The error bars indicate the interquartile ranges (IQR). The x-axe indicates the different imaging modality. The y-axe gives the score each surgeon assigned to the different modality per case. **CI** = conventional imaging (MRI and/or CT), **3D** = 3D print, **AR =** Augmented Reality.

© 2019 Wellens LM et al. *JAMA Network Open*.
